# Coffee and Lower Risk of Type 2 Diabetes: Arguments for a Causal Relationship

**DOI:** 10.3390/nu13041144

**Published:** 2021-03-31

**Authors:** Hubert Kolb, Stephan Martin, Kerstin Kempf

**Affiliations:** 1 Faculty of Medicine, University of Duesseldorf, Moorenstr. 5, 40225 Duesseldorf, Germany; hubert.kolb@hhu.de (H.K.); stephan.martin@uni-duesseldorf.de (S.M.); 2 West-German Centre of Diabetes and Health, Duesseldorf Catholic Hospital Group, Hohensandweg 37, 40591 Duesseldorf, Germany

**Keywords:** coffee, diabetes, caffeine, chlorogenic, acids, hepatosteatosis, beta cells, hormesis, Nrf2

## Abstract

Prospective epidemiological studies concur in an association between habitual coffee consumption and a lower risk of type 2 diabetes. Several aspects of these studies support a cause–effect relationship. There is a dependency on daily coffee dose. Study outcomes are similar in different regions of the world, show no differences between sexes, between obese versus lean, young versus old, smokers versus nonsmokers, regardless of the number of confounders adjusted for. Randomized controlled intervention trials did not find a consistent impact of drinking coffee on acute metabolic control, except for effects of caffeine. Therefore, lowering of diabetes risk by coffee consumption does not involve an acute effect on the post-meal course of blood glucose, insulin or insulin resistance. Several studies in animals and humans find that the ingestion of coffee phytochemicals induces an adaptive cellular response characterized by upregulation and de novo synthesis of enzymes involved in cell defense and repair. A key regulator is the nuclear factor erythroid 2-related factor 2 (Nrf2) in association with the aryl hydrocarbon receptor, AMP-activated kinase and sirtuins. One major site of coffee actions appears to be the liver, causing improved fat oxidation and lower risk of steatosis. Another major effect of coffee intake is preservation of functional beta cell mass via enhanced mitochondrial function, lower endoplasmic reticulum stress and prevention or clearance of aggregates of misfolded proinsulin or amylin. Long-term preservation of proper liver and beta cell function may account for the association of habitual coffee drinking with a lower risk of type 2 diabetes, rather than acute improvement of metabolic control.

## 1. Introduction

The perception of coffee has experienced a remarkable transition from a stimulant drink which may stress your cardiovascular system to a beverage that is good for your health. The latter view is derived from a large number of prospective cohort studies which observed an association of socioeconomic or lifestyle factors including habitual coffee consumption and clinical outcomes including type 2 diabetes mellitus, non-alcoholic fatty liver disease, liver cancer, gout, kidney stones and Parkinson’s disease [1].

In this paper, we discuss the association of habitual coffee consumption and a lower risk of type 2 diabetes. In addition to epidemiological studies, data are available from a considerable number of randomized short-term intervention trials with metabolic endpoints, and from Mendelian randomization studies. Finally, we consider that coffee constituents appear to exert similar molecular effects at the cellular level as reported for phytochemicals of other dietary plants. From these data, a picture emerges how coffee consumption promotes resistance to the development of type 2 diabetes.

This paper is a narrative review and commentary based on a survey of all papers listed by PubMed for the search items coffee, caffeine or chlorogenic acid, in combination with the items diabetes, glucose tolerance or insulin resistance, respectively. Other papers were retrieved from the reference list of reviews published on coffee, caffeine or chlorogenic acid versus metabolic endpoints. To the best of our knowledge, we included and dis-cussed all published human trials of coffee consumption versus metabolic endpoints. For the discussion of a possible molecular mechanism, we conducted an additional search in PubMed with the items coffee/caffeine/chlorogenic acid in combination with the items Nrf2/anti-oxidative/anti-inflammatory, respectively.

## 2. Epidemiological Studies

Although epidemiological studies cannot prove a causal relationship all recent meta-analyses considered it probable that coffee consumption lowers the risk of type 2 diabetes [1,2,3,4]. Up to 30 prospective cohort studies were included in the meta-analyses, with more than a million participants, and more than 50,000 cases during a follow-up period of up to 24 years. When comparing the cohorts with the highest category of coffee consumption (median around 5 cups per day) versus no coffee consumption, the pooled relative risk of type 2 diabetes was around 0.7. The risk decreased by ~6 % for each additional cup of coffee consumed, and this relationship was fairly linear except for a possible flattening of the curve for the small subgroups drinking more than six cups per day.

Observational studies suffer from different characteristics of subgroups drinking none, little or much coffee. Consumption of none or little coffee may be due to intestinal intolerance, religious reasons or simply dislike or prejudice. It is virtually impossible to correct for this type of confounding. The number of confounders considered in the various studies is limited (listed in [3]). For instance, one important confounder that is not recognized in any of the studies, is brushing of teeth. This may happen more often in coffee consumers to prevent staining of teeth, and at the same time it lowers the risk of periodontitis, a major risk factor of low grade systemic inflammation and type 2 diabetes [5]. Other diabetes risk factors not considered as confounders include exposure to traffic noise or fine dust.

Nonetheless, it seems improbable that residual confounding accounts for the association of coffee consumption with a lower risk of type 2 diabetes, for the following reasons. (i) Results of prospective cohort studies are surprisingly similar in different regions of the world (Europe, USA, Asia) although lifestyle and cultural background differ. (ii) There is no significant difference in outcome for men and women. (iii) Sub-analyses for study participants with obesity (body mass index > 25), with age above 50 years or for non-smokers also observed an inverse association of coffee consumption and risk of type 2 diabetes [6,7]. (iv) A similar inverse association was reported for drinking unfiltered boiled coffee as well as for filtered coffee [8,9]. (v) It is difficult to consider a confounder that is responsible for the linear relationship between quantity of coffee consumed and risk of diabetes. (vi) Studies that correct for only six possible confounders report similar outcomes as studies that considered 15 or more possible confounders (data from [3]).

Another epidemiological approach is to correlate changes in coffee consumption pattern with diabetes outcome. Three large prospective studies in the USA of 16–20 years duration and documentation of dietary habits every four years were analyzed for changes in coffee consumption [10]. An increase of daily coffee consumption by > 1 cup (median 1.69 cups) was associated with an 11% lower relative risk of type 2 diabetes compared to those who made no changes. Conversely, a decrease in consumption by > 1 cup (median 2 cups) was associated with a 17% higher risk of type 2 diabetes. In order to minimize the chance of reverse causation, the subgroups were adjusted for baseline differences and for later changes that may have caused altered coffee consumption, such as increased/decreased physical activity or cardiovascular disease. Interestingly, changes in tea consumption were not associated with changes in diabetes risk.

Taken together, epidemiological studies concur on an inverse association of habitual coffee consumption with risk of type 2 diabetes. The association with diabetes risk is as robust as that of other lifestyle factors not tested in randomized long-term trials such as physical activity, sitting time, sleep duration, smoking or exposure to traffic noise and fine dust [11].

## 3. Mendelian Randomization Studies

Genome-wide association studies have identified several genetic variants seen more often in coffee drinkers than non-drinkers [12,13,14,15]. The strongest effect size is seen for gene variants that are involved in caffeine metabolism. Heavy coffee consumption is associated with gene variants allowing faster caffeine breakdown. Many of the variants associated with coffee consumption are also associated with other traits and therefore might modulate the risk of type 2 diabetes via other pathways than promoting coffee consumption [16].

Several Mendelian randomization studies have searched for a higher diabetes risk in carriers of gene variants promoting coffee consumption, but results remained inconclusive or did not show a causal link with incident type 2 diabetes or other health outcomes [15,16,17]. One probable reason is that the genetic tolerance of higher caffeine intake may promote consumption of any caffeine containing drink including black or green tea, but consumption of the latter is not consistently associated with lower diabetes risk [18,19]. Furthermore, genetic variants account for less than half a cup of coffee consumed, i.e., about 40 mg caffeine [12] which may be too small to account for different diabetes risk. When the same large cohort (DIAbetes Genetics Replication And Meta-analysis (DIAGRAM)) was analyzed for partially different sets of genetic variants found associated with coffee or caffeine consumption in previous cohorts, one analysis did not observe an association of genetically determined caffeine consumption with diabetes risk [15] whereas another analysis found a significant link between diabetes risk and genes variants promoting caffeine or coffee consumption, respectively [20].

As described above, changes in coffee consumption in individuals followed in prospective studies was accompanied with an inverse change of diabetes risk while this was not the case for tea [10]. In this setting, genetic characteristics of participants do not differ before and after modification of coffee consumption habits.

We conclude that there is no major influence of individual genetic characteristics associated with coffee consumption regarding the risk of type 2 diabetes. The responsible molecular mechanism involved seems directly linked to coffee consumption and not dependent on genetic variants promoting coffee/caffeine consumption.

## 4. No Acute Impact on Metabolic Control: Caffeinated Coffee

It has been difficult to identify acute metabolic effects of coffee consumption in randomized controlled intervention trials except for effects of caffeine. The major pharmacological activity of caffeine is inhibition of adenosine receptors and modulation of the purinergic system because of the molecular similarity to adenosine [4,21]. As a consequence, adenosine-mediated vasodilation and many additional physiological functions of adenosine, notably in the brain, are antagonized. Tolerance to these caffeine effects develops within a week of daily coffee consumption but may be incomplete and lost as rapidly during caffeine abstinence [4]. Trials of caffeinated coffee or caffeine usually have a run-in phase of little or no caffeine consumption. Therefore, tolerance to caffeine is decreased and acute effects of caffeine become recognizable but disappear again after a longer period of coffee/caffeine intake. Acute effects of coffee or caffeine include modestly reduced appetite, an increased metabolic rate/thermogenesis and reduced insulin sensitivity [22,23,24,25,26,27,28,29,30,31,32]. It is probable that the enhanced activity of the sympathetic nervous system and increased epinephrine release is a major contributor to these acute metabolic responses [4,23,24].

The acute metabolic effects of caffeinated coffee or caffeine did not persist after longer periods of coffee consumption. A 24-week trial with 126 overweight Asian participants reported no significant change of insulin sensitivity after daily consumption of 4 cups of caffeinated coffee compared to a coffee-like placebo drink. There was also no difference in fasting glucose [33]. A trial in 45 overweight Japanese participants with mild-to-moderate elevation of fasting blood glucose found no change in oral glucose tolerance measures nor of insulin sensitivity after 8 weeks of daily consumption of 5 cups of caffeinated or decaffeinated coffee compared to water. After 16 weeks, the post load glucose levels were mildly decreased in the caffeinated coffee group only [34]. A trial in 45 overweight North American participants reported no change in insulin sensitivity and oral glucose tolerance after consumption of caffeinated or decaffeinated coffee for 8 weeks, compared with water [35]. The only long-term metabolic effects of caffeinated coffee consumption compared with control was a ~4 % decrease of fat mass (after 24 weeks [33]) or a ~2 cm reduction in waist circumference (after 16 weeks [34]).

Taken together, habitual consumption of caffeinated coffee does not impact metabolic control of glucose and insulin levels but may have a mild effect in favor of caffeine-mediated body fat/visceral fat loss. In the long term, these changes may be sufficient for lowering the risk of type 2 diabetes, because lifestyle intervention trials have shown that modest lowering of body weight by 5%–7% is already associated with a decreased diabetes rate [36]. However, several prospective cohort studies compared outcomes for caffeinated and decaffeinated coffee and report similarly decreased diabetes risk for both types of coffee. Meta-analyses revealed a relative risk of type 2 diabetes for each cup-per-day increase in coffee consumption of 7%–9% for caffeinated coffee and 6 % for decaffeinated coffee, difference not significant [3,37].

It, therefore, may be concluded that the presence of caffeine in coffee and the accompanying modest decrease in body fat is not essential for its apparent diabetes-protective effect although there may be a minor contribution. Other constituents of coffee must be viewed as candidates for lowering the risk of type 2 diabetes.

## 5. No Acute Impact on Metabolic Control: Decaffeinated Coffee

Decaffeinated coffee contains only very small amounts of caffeine [38] so that beneficial effects of other coffee constituents on metabolic control may become apparent. More than 10 randomized controlled trials of acute effects of decaffeinated coffee consumption on the metabolic response to subsequent ingestion of glucose or a high glycemic index meal have been performed, without providing a clear message.

Three trials did not report on a control group with water or placebo fluid consumption instead of coffee, and therefore, do not provide sound evidence [28,39,40]. Three further trials did not notice an impact of decaffeinated coffee consumption on the course of blood glucose or insulin levels in response to a glucose load [41,42,43]. One trial observed a transiently increased glucose level in response to a high glycemic index meal but no difference in overall glucose or insulin levels when comparing decaffeinated coffee and water [44]. A few trials measured incretin levels and/or insulin sensitivity and reported no or mild and not consistent changes in the decaffeinated coffee groups. These possible effects were not reflected by an impact on blood glucose or insulin kinetics [41,43,45]. Conversely, one trial reported transiently increased blood glucose and persistently elevated insulin levels during an oral glucose tolerance test after consuming decaffeinated coffee compared with water, but no impact on insulin sensitivity was seen [46].

After 8 weeks of daily drinking decaffeinated coffee (*n* = 14) there was no difference to the water control (*n* = 15) with respect to the glucose and insulin response to an oral glucose load, or with regard to insulin resistance, in healthy, middle-aged and overweight persons [35]. Another trial also reported no impact on oral glucose tolerance after 16 weeks of consumption of decaffeinated coffee (*n* = 17) versus water (*n* = 13) [34].

The trials usually included 10–17 participants, and these were young, metabolically healthy and lean persons (overweight middle-aged participants in only one study). Several endpoints were analyzed at different points of time, usually without correction for the high number of statistical tests performed. Taken together, the trials do not provide evidence for an acute effect on metabolic control after consumption decaffeinated coffee. Long-term trials would be required to analyze for a delayed impact of metabolic control and the prevention of metabolic deterioration or with diabetes as clinical endpoint. Such trials currently do not appear feasible.

A summary of conclusions from observational and randomized controlled studies of coffee consumption is given in Box 1.

Box 1 **Findings of observational and randomized studies**

- Prospective cohort studies find a lower risk of type 2 diabetes associated with habitual coffee consumption.
- The association with diabetes risk is dose dependent, seen world-wide and in both sexes, and is also found for decaffeinated coffee.
- The association with diabetes risk is seen regardless of the number of potential confounders adjusted for.
- Changes of coffee consumption over time are accompanied by a change of diabetes risk.
- Mendelian randomization studies do not provide consistent results on the association of diabetes risk with a genetic background favoring caffeine/coffee consumption. However, the genetic effect size on caffeine/coffee intake is modest.
- Randomized controlled trials do not observe a consistent acute impact of coffee consumption on metabolic control except for some beneficial effects of caffeine on appetite and body fat mass. Long term trials with diabetes as endpoint are not feasible.

## 6. Candidate Mechanisms for a Delayed Impact on Metabolic Control: Metabolomics

Because randomized controlled trials of short duration failed to uncover a consistent caffeine-independent modulatory effect of coffee consumption on systemic insulin or glucose levels after a meal or glucose challenge, other physiological responses appear to be relevant. These include delayed impact on metabolic control and prevention of metabolic deterioration.

One approach to identify relevant physiological responses to coffee is to screen for an impact on a wide variety of circulating components of metabolism, other than insulin and glucose. A recent analysis of the Nurses’ Health Study II identified three cholesteryl esters associated with coffee consumption and lower diabetes risk. Conversely, five diacylglycerols and seven triacylglycerols showed negative associations with coffee-related diabetes risk factors [47]. A lipidomic analysis revealed a decrease of most lipid metabolites, including cholesteryl ester and triacylglycerols. How these changes related to diabetes risk was not studied [48]. Analyses of the metabolomic response to coffee consumption have observed that many different metabolic pathways are affected ranging from steroid synthesis to amino acid metabolism [49,50,51,52]. Taken together, metabolomic analyses have identified many changes caused by coffee consumption, but the possible relationship to a lower diabetes risk remains unresolved.

## 7. Candidate Mechanisms for a Delayed Impact on Metabolic Control: Known Actions of Phytochemicals

Many phenolic phytochemicals exhibit concentration-dependent toxic properties which include DNA damage, mutagenesis, carcinogenesis and cell death. Organs affected include the liver, intestine and kidney. Tumor cells appear to be more susceptible than normal cells [53,54,55,56,57,58,59,60,61].

The studies have been performed with high doses of phenolic compounds from foods like coffee, tea or other edible plants. Despite these potentially toxic properties, vegetarian food is well tolerated by the human organism. The reason is the subtoxic dose of phytochemicals taken up with our daily diet. Postprandial blood concentrations of major dietary phenolics may reach a few µmol/l but usually are lower [62,63]. Cytotoxicity towards healthy cells is observed at concentrations of 10 µmol/l or higher, depending on the phenolic compound and cell type tested. In humans, at present the only phenolic compounds for which an upper limit of consumption is recommended, because of possible hepatotoxicity, are green tea catechins [64].

Coffee appears to be a safe beverage in that regard except for high intakes of caffeine, especially during pregnancy [65]. Randomized controlled trials of moderate coffee consumption for several months have not identified detrimental physiological responses [33,35,66]. We excluded caffeine from the following discussion of antidiabetic coffee effects because epidemiological studies find decaffeinated coffee almost as strongly associated with a lower risk for type 2 diabetes as caffeinated coffee.

After consumption of one cup of coffee, peak blood concentrations of chlorogenic acid metabolites were about 1 µmol/l, whereas pyridine derivatives trigonelline and 1-methylpyridinium reached peak concentrations of 6 and 1 µmol/l. Peak concentrations are below the micromolar range for all other phytochemicals in coffee, except for caffeine [67]. Even at these low concentrations, coffee phytochemicals and their metabolites interact with many components of cells and organs. Cellular targets for physical binding of phytochemicals include the complex of nuclear factor erythroid 2-related factor 2 (Nrf2) and Kelch-like ECH-associating protein-1 (Keap1) [68,69,70,71], the aryl hydrocarbon receptor (AHR) [72,73,74,75], protein disulfide isomerase3 [76], 3-hydroxy-3-methylglutaryl-coenzyme A reductase [77], protein kinase B (AKT) [78], glutathione S-transferase pi isoform-1 [79], vascular endothelial growth factor receptor [80], PPARγ [81], amyloid forming peptides [55,82,83,84,85,86,87], basic proline-rich protein in saliva [88], human serum albumin [89,90] or low density lipoprotein [91].

Most of these interactions are weak at micromolar or submicromolar concentrations but randomized controlled trials of coffee consumption in humans report one major cellular response, the increased expression of proteins involved in the defense against free radicals, xenobiotics or UV irradiation, improved cell regeneration, DNA repair and cell survival, as well as dampening of pro-inflammatory activities [92]. These reactions primarily occur by activation of the Nrf2/Keap1 system and the AHR, and they can be considered as an adaptive response to the mild chemical stress mediated by phytochemicals of coffee or other plants. Keap1 and AHR are sensors for xenobiotics that target cysteine residues (Keap1) or a hydrophobic binding region (AHR) for electrophilic/hydrophobic compounds among phytochemicals which initiates a cascade of events resulting in upregulation of cellular defense mechanisms.

As described in detail previously [92], Nrf2 is a nuclear factor which binds to characteristic DNA sequences (electrophile/antioxidant response elements) in the 5′-upstream regions of a large number of genes involved in cell defense, giving rise to increased gene transcription. Cytoprotective actions include the expression of antioxidant enzymes and xenobiotic detoxifying enzymes, stress proteins, increased turnover of misfolded proteins, improved mitochondrial biogenesis and function, decreased expression of the immunoregulatory nuclear factor NFkB and of pro-inflammatory molecules like tumor necrosis factor-α or the NLRP3 (NOD-like receptor family, pyrin domain containing 3) inflammasome.

There is continuous de novo synthesis of Nrf2, most of which is captured by cytosolic Keap1 and channeled to proteasomal degradation. Keap1 is highly sensitive to an electrophile attack by phytochemicals because of its 17 cysteine residues and readily loses its ability to recycle Nrf2 and prevent its translocation to the nucleus where it targets the DNA sequences described above. Phytochemicals may also indirectly activate the Nrf2/Keap1 system by increasing the intracellular levels of oxygen radical species or nitric oxide all of which target cysteine residues of Keap 1. Free oxygen radical species including H_2_O_2_ may be either generated directly by phenolics extra- or intracellularly, in the presence of Cu(II) or Fe(III) ions [55,56,60,93,94], or may come from mitochondria [95,96,97,98,99,100].

Electrophilic phytochemicals including those of coffee may also target the aryl hydrocarbon receptor [73,74]. This nuclear factor is stabilized in the cytoplasm by a chaperone complex and kept in an inactive state. Upon binding of a suitable electrophilic ligand AHR and some components of the cytoplasmic complex translocate to the nucleus where they promote the expression of genes involved in detoxifying xenobiotics by binding to xenobiotic response elements (XRE). In addition, AHR interacts with other transcription factors involved in various cellular regulation circuits [101]. Activation of AHR activates Nrf2 by several mechanisms such as increased Nrf2 gene expression or reactive oxygen species (ROS) production from AHR induced cytochrome P450 1A enzyme activity [102,103].

While the Nrf2/Keap1 and AHR systems respond to increased levels of electrophiles or free radicals, another approach of sensing danger in cells is monitoring the energy level. The response to phytochemical stress is expected to affect cellular energy reserve. A low energy state is characterized by low ATP/AMP and ADP/ATP ratios as well as low glucose levels. This condition leads to the activation of AMP-activated protein kinase (AMPK). This group of enzymes increase energy production and decreases energy uses by phosphorylation and promoting acetylation of many metabolic enzymes, histones and transcription factors. There is also activation of histone deacetylases of the sirtuin family by provision of NAD^+^ which promotes mitochondrial function, autophagy and a gene expression pattern supporting cell survival during various forms of stress including those mediated by phytochemicals [104,105,106]. The activation of AMPK and sirtuin 1 in cells exposed to phytochemicals is associated with the activation of the Nrf2/Keap1-system, forming a regulatory network [78,107].

The possible health effect of coffee is supported by findings that several different components of coffee are able to activate the Nrf2/Keap1 or AHR systems in isolated cells, animals and humans. Chlorogenic acid and its degradation product caffeic acid are strong activators of Nrf2 activity [108,109,110] as also reported for melanoidins [111], kahweol and cafestol [112,113,114]. Another coffee constituent, trigonelline, inhibits Nrf2 activation possibly by interfering with the epidermal growth factor signaling pathway [108,115]. Roasting of coffee causes thermal degradation of trigonelline and the accumulation of degradation products nicotinic acid and pyridine derivatives like N-methylpyridinium and 1,2-dimethylpyridinium [63,67]. The pyridine derivatives are potent activators or Nrf2 and AHR dependent gene expression [108,116]. For N-methylpyridinium, a concentration of 0.1µmol/l was found sufficient for activating Nrf2 [108].

**Figure 1:**
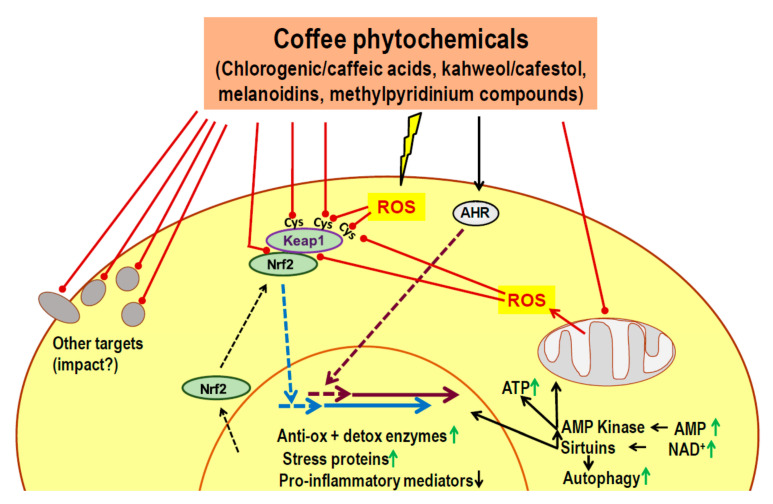
Activation of the nuclear factor erythroid 2-related factor 2 (Nrf2)/Kelch-like ECH-associating protein-1 (Nrf2/Keap1) system and of hydrophobic binding region (AHR) by phytochemicals in roasted (decaffeinated) coffee. Major coffee constituents exhibit binding properties to a number of cellular targets. Consistent physiological responses are elicited by direct (electrophilic) binding to cysteine residues of Keap1 and to AHR, causing the translocation of Nrf2 and AHR, respectively, to the nucleus and the increased expression of genes involved in cell protective activities. Furthermore, there is increased production of reactive oxygen species (ROS), either directly generated from reactive phenolics or indirectly via perturbation of mitochondrial function. Cysteine residues of Keap1 act as sensors of oxidative stress. The resulting modification of Keap1 allows Nrf2 to translocate to the nucleus. Cell stress may result in a lower energy level and increased concentrations of adenosine monophosphate (AMP) and (nicotinamide adenine dinucleotide) NAD^+^ which causes the activation of AMP-activated kinase and of sirtuins, respectively. AMPK lowers anabolic and increases catabolic activities for increasing (adenosine triphosphate) ATP levels. Sirtuins deacetylate histones and other targets leading to improved mitochondrial function, increased autophagy and the expression of genes mediating improved cell survival during periods of stress. ↑, upregulation; ↓ downregulation

Taken together, there is evidence that consumption of coffee activates a cell protective, Nrf2 and AHR dependent cell response in a systemic manner [116] which includes the gut lining [111] (Figure 1). As reviewed previously, this physiological response to coffee drinking is analogous to the response following the ingestion of phytochemicals from other plants [92]. A special role of coffee in the uptake of dietary phytochemicals follows from the observation that coffee is the major dietary source of phenolics, providing about 70% in the diet and even surpassing green tea phenolics even in Japan [117,118,119].

The upregulation of cell protective regulatory circuits in response to the ingestion of coffee appears to occur in many organs of the body, as judged from analyses of liver, hepatocytes, stomach, blood lymphocytes, endothelial cells, muscle cells and the small intestine [73,109,110,111,113,116,120,121,122,123,124,125]. The available data suggest that a Nrf2-dependent mechanism for the antidiabetic action of coffee may focus on the liver and the beta cell.

Coffee and liver: In vivo imaging using a luciferase-reporter gene system in rodents showed that the liver is the main organ responding to coffee ingestion with the expression of Nrf2-regulated genes [121]. Feeding of decaffeinated coffee upregulated liver expression of endoplasmic reticulum and mitochondrial chaperones as well as antioxidative enzymes [122,123] and concomitantly prevented or mitigated the development of nonalcoholic fatty liver disease (NAFLD) during a high fat diet. Both caffeine and phenolic coffee constituents like chlorogenic acids contribute to these effects which include reduced oxidative and endoplasmic reticulum stress, increased autophagy/lipophagy, fatty acid ß-oxidation and lipolysis, in conjunction with improved mitochondrial activity and decreased levels of liver transaminases [126,127,128]. Deficient handling of saturated fats by mitochondria is considered a key factor in the development of liver steatosis [129]. There is only limited data on the modulation of the microbiota by coffee constituents except for increased production of short chain fatty acids and an improved intestinal barrier function. The latter may contribute to the prevention of NAFLD by lowering exposure of the liver to pro-inflammatory microbial components [122,130,131].

In accordance with the animal experiments, prospective studies of coffee consumption versus incident NAFLD or liver cirrhosis in humans reported an inverse relationship [132,133]. There is a non-linear dose-dependency, and a meta-analysis reported a significant negative association at >3 cups of coffee per day [132]. It, therefore, seems probable that habitual coffee consumption leads to more effective hepatic handling of lipids which, together with less leakage of pro-inflammatory compounds from the microbiota decreases the risk of NAFLD, cirrhosis and type 2 diabetes.

Coffee and beta cells: Loss of functional beta cell mass in relation to insulin requirements is the critical process causing type 2 diabetes [134]. The lower incidence of type 2 diabetes in habitual coffee drinkers therefore likely involves better preservation of beta cell function.

Prospective studies indicate that increased insulin secretion and hyperinsulinemia precedes type 2 diabetes in most people [135,136]. The high synthesis rate of insulin is associated with increased production of ROS, mostly from mitochondria, with endoplasmic stress, accumulation of improper folded peptides and subsequent loss of endocrine cell function and eventually cell death [137,138,139,140,141].

Upregulation of Nrf2 activity in beta cells by phytochemicals counteracts the damaging effects of glucolipotoxicity (in vitro) or a high fat diet (in vivo) [142,143,144,145,146]. In beta cells under oxidative stress Nrf2 activation helps to lower the level of ROS and increase the production of NADPH to a range required for physiological glucose stimulated insulin secretion [147]. Proof of a causal role of Nrf2 for preservation of functional beta cells comes from studies of genetic manipulation of Nrf2 activation. A knockdown of the *Nrf2* gene substantially reduced the defense response of beta cells towards oxidative and nitrosative damage, disturbed mitochondrial function and diminished glucose-stimulated beta cell proliferation [148,149]. Conversely, knockdown of the *keap 1* gene led to activation of Nrf2 which largely prevented diabetes development and beta cell loss in the spontaneously diabetic *db/db* mice, and also prevented diabetes development in a high calorie-diet model [150]. Preservation of islet histology and less steatohepatitis was also seen in a mouse model of spontaneous metabolic syndrome after adding coffee to the drinking water [151].

Coffee phytochemicals such as 5-O-caffeoylquinic acid, pyrocatechol and melanoidins as well as polyphenols from other plants can preserve cell function in periods of metabolic stress also by interfering with the aggregation of misfolded proteins into amyloids [85,86,87,152,153,154]. Aggregation of misfolded proinsulin or amylin molecules in beta cells is known to precede the onset of type diabetes and to impair cell functions [155].

**Figure 2:**
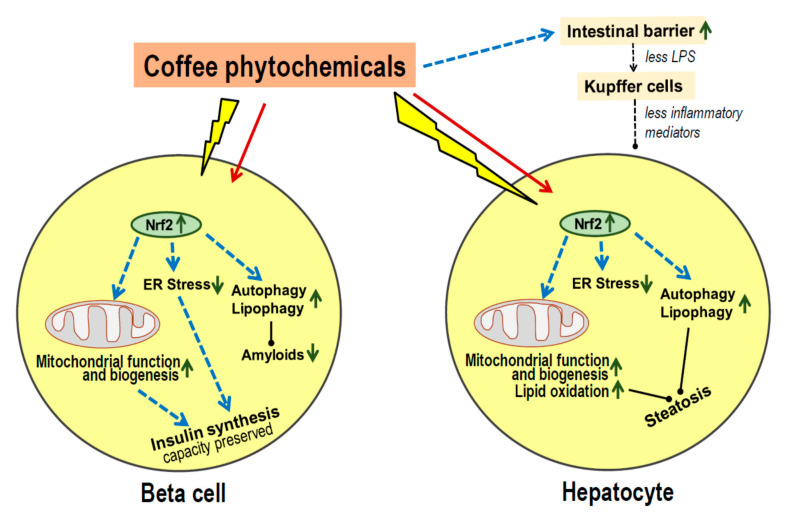
Protective action of coffee on metabolic stress in islet beta cells and hepatocytes. Exposure to coffee or its major constituents leads to activation of nuclear factor erythroid 2-related factor 2 (Nrf2), in vitro as well as in vivo. Enhanced expression of Nrf2 regulated genes leads to improved mitochondrial function and oxidation of lipids, and to mitigating of endoplasmic reticulum (ER) stress in cells during periods of increased peptide synthesis. In beta cells, the insulin production capacity is further preserved by eliminating aggregates of misfolded proinsulin or amylin by increased autophagy, and by prevention of aggregation by binding of coffee phytochemicals to hydrophobic surface regions exposed by misfolded peptides. In hepatocytes, metabolic activity is preserved and steatosis prevented by improved mitochondrial beta-oxidation of fatty acids, increased autophagy for lipid disposal and less inflammatory stress from activated Kupffer cells because of less lipopolysaccharide (LPS) leakage from the colon. ↑, upregulation; ↓ downregulation

Taken together, coffee phytochemicals can sustain beta cell function and survival (Figure 2). There is activation of Nrf2 leading to sustained upregulation of anti-oxidative defense, improved mitochondrial function and biogenesis and prevention of cell damage during periods of high insulin secretion in the prediabetic state. Further, several coffee constituents interfere with the aggregation of misfolded proinsulin and amylin observed in beta cells during periods of high biosynthetic activity. These effects of coffee components fit with long-term preservation of functional beta cell mass rather than acute improvement of insulin secretion, and are therefore, in line with the lack of an acute metabolic effect of (decaffeinated) coffee.

## 8. Conclusions

A summary of conclusions is given in Box 2.

Box 2 **Key Messages**

- Prospective epidemiological studies find a robust association of habitual coffee consumption (caffeinated or decaffeinated) with a lower risk of type 2 diabetes.
- Results of Mendelian randomization studies remain inconclusive, possibly because of a small effect size.
- Randomized controlled trials do not show a consistent effect of coffee intake on acute metabolic control, except for some effects of caffeine.
- Metabolomic analyses also do not provide a clear picture how coffee might modulate metabolic control.
- Phytochemicals of coffee or other dietary plants are known to induce an adaptive cell response characterized by activation of Nrf2, AHR, AMPK and sirtuins.
- Most coffee phytochemicals and metabolites accumulate in the liver. The resulting Nrf2-dependent toxic stress response improves mitochondrial function, lipid oxidation and reduces the risk of steatosis.
- Data on modulation of the gut microbiota are scarce, but there seems to be an improved intestinal barrier function which will contribute to the prevention of steatosis.
- Coffee phytochemicals support the preservation of pancreatic beta cell function via Nrf2-mediated resistance to cell damage during periods of high insulin secretion. In addition, coffee constituents directly interact with misfolded peptides and prevent the formation of cell-toxic amyloids.
- Long-term effects of habitual coffee consumption appears to maintain proper function of liver and beta cells rather than improve acute metabolic control.

Although epidemiological studies consistently find an association of habitual coffee consumption formal proof of a cause-effect relationship is lacking. However, several aspects of observational prospective studies strongly argue against a major influence of residual confounding and support an antidiabetic effect of coffee. Results of prospective observational studies are surprisingly similar for different regions of the world, including coffee-dose dependency, no difference between male versus female, obese versus lean, younger versus older study participants, regardless of the number of confounders adjusted for. Analyses for habitual consumption of decaffeinated coffee yielded similar results as for caffeinated coffee [3,6,7]. Changes in coffee consumption over time correlated with changes in diabetes risk [10]. Results of Mendelian randomization studies did not help to prove or disprove causality because they were not consistent and suffered from a small effect size [12,15,16,17,20].

Short-term randomized controlled trials comparing consumption of coffee versus water or a placebo drink did not provide clues about the possible mechanism of diabetes prevention. Aside from acute consequences of caffeine intake there were no consistent effects on diabetes-relevant metabolic parameters such as the insulin and glucose response to a glucose load or to a meal. Insulin sensitivity was not modulated (see detailed description above). We conclude that a diabetes-preventive effect of coffee does not bear on acute metabolic responses but must exhibit a delayed impact on metabolic control and prevent metabolic deterioration.

One consistent biochemical response to the consumption of coffee phytochemicals is an improved antioxidative defense in animals and humans, such as elevated levels of glutathione, catalase and superoxide dismutase [123,156,157]. Mechanistic studies revealed a key role of the activation of Nrf2 and inactivation of Keap1, complemented by engaging AHR, AMPK and sirtuins [92].

The liver is the primary organ for the accumulation of coffee phytochemicals and metabolites [121]. The ensuing protective Nrf2-dependent cell response involves improved mitochondrial function and ß-oxidation of fatty acids which prevents liver steatosis in rodents fed a high fat diet [126,127,128]. Liver function is further supported by an increased intestinal barrier function, probably mediated by a prebiotic effect of coffee constituents on the colon microbiota [122,130,131]. Other modulatory effects of coffee on gut functions that might be relevant for diabetes prevention have not been reported.

Besides the liver, the other prominent target for a long-term diabetes-preventive effect of coffee consumption is probably the pancreatic beta cell. In the prediabetic period, beta cells are exposed to metabolic stress associated with obesity and insulin resistance. Glucolipotoxicity involves deficient mitochondrial function and endoplasmic reticulum stress which leads to beta cell dysfunction, dedifferentiation or death [134,135,136]. Activation of the Nrf2 system appears to counteract these cell damaging processes and help maintain proper cell functions. Nrf2 therefore may be considered the “master and captain of beta cell fate” [147]. In beta cells synthesizing hormones at high rate, aggregates of misfolded proinsulin or amylin may accumulate and contribute to cell toxicity [141,155]. Coffee phytochemicals can directly bind to misfolded peptides which prevents or reverses amyloid formation [152,153].

Taken together, habitual coffee consumption may lower the risk of type 2 diabetes by preventing the deterioration of liver and beta cell function during chronic metabolic stress preceding the manifestation of overt diabetes.

## References

[1] Poole R., Kennedy O.J., Roderick P., Fallowfield J.A., Hayes P.C., Parkes J. (2017). Coffee consumption and health: Umbrella review of meta-analyses of multiple health outcomes. BMJ.

[2] Grosso G., Godos J., Galvano F., Giovannucci E.L. (2017). Coffee, Caffeine, and Health Outcomes: An Umbrella Review. Annu. Rev. Nutr..

[3] Carlstrom M., Larsson S.C. (2018). Coffee consumption and reduced risk of developing type 2 diabetes: A systematic review with meta-analysis. Nutr. Rev..

[4] Van Dam R.M., Hu F.B., Willett W.C. (2020). Coffee, Caffeine, and Health. N. Engl. J. Med..

[5] Wu C.Z., Yuan Y.H., Liu H.H., Li S.S., Zhang B.W., Chen W., An Z.J., Chen S.Y., Wu Y.Z., Han B. (2020). Epidemiologic relationship between periodontitis and type 2 diabetes mellitus. BMC Oral Health.

[6] Van Dam R.M., Hu F.B. (2005). Coffee consumption and risk of type 2 diabetes: A systematic review. JAMA.

[7] Jiang X., Zhang D., Jiang W. (2014). Coffee and caffeine intake and incidence of type 2 diabetes mellitus: A meta-analysis of prospective studies. Eur. J. Nutr..

[8] Hjellvik V., Tverdal A., Strom H. (2011). Boiled coffee intake and subsequent risk for type 2 diabetes. Epidemiology.

[9] Tuomilehto J., Hu G., Bidel S., Lindstrom J., Jousilahti P. (2004). Coffee consumption and risk of type 2 diabetes mellitus among middle-aged Finnish men and women. JAMA.

[10] Bhupathiraju S.N., Pan A., Manson J.E., Willett W.C., van Dam R.M., Hu F.B. (2014). Changes in coffee intake and subsequent risk of type 2 diabetes: Three large cohorts of US men and women. Diabetologia.

[11] Kolb H., Martin S. (2017). Environmental/lifestyle factors in the pathogenesis and prevention of type 2 diabetes. BMC Med..

[12] Cornelis M.C., Monda K.L., Yu K., Paynter N., Azzato E.M., Bennett S.N., Berndt S.I., Boerwinkle E., Chanock S., Chatterjee N. (2011). Genome-wide meta-analysis identifies regions on 7p21 (AHR) and 15q24 (CYP1A2) as determinants of habitual caffeine consumption. PLoS Genet..

[13] Amin N., Byrne E., Johnson J., Chenevix-Trench G., Walter S., Nolte I.M., Vink J.M., Rawal R., Mangino M., Teumer A. (2012). Genome-wide association analysis of coffee drinking suggests association with CYP1A1/CYP1A2 and NRCAM. Mol. Psychiatry.

[14] Cornelis M.C., Byrne E.M., Esko T., Nalls M.A., Ganna A., Paynter N., Monda K.L., Amin N., Fischer K., Renstrom F. (2015). Genome-wide meta-analysis identifies six novel loci associated with habitual coffee consumption. Mol. Psychiatry.

[15] Said M.A., van de Vegte Y.J., Verweij N., van der Harst P. (2020). Associations of Observational and Genetically Determined Caffeine Intake With Coronary Artery Disease and Diabetes Mellitus. J. Am. Heart Assoc..

[16] Cornelis M.C., Munafo M.R. (2018). Mendelian Randomization Studies of Coffee and Caffeine Consumption. Nutrients.

[17] Nicolopoulos K., Mulugeta A., Zhou A., Hypponen E. (2020). Association between habitual coffee consumption and multiple disease outcomes: A Mendelian randomisation phenome-wide association study in the UK Biobank. Clin. Nutr..

[18] Yang J., Mao Q.X., Xu H.X., Ma X., Zeng C.Y. (2014). Tea consumption and risk of type 2 diabetes mellitus: A systematic review and meta-analysis update. BMJ Open.

[19] Liu X., Xu W., Cai H., Gao Y.T., Li H., Ji B.T., Shu X., Wang T., Gerszten R.E., Zheng W. (2018). Green tea consumption and risk of type 2 diabetes in Chinese adults: The Shanghai Women’s Health Study and the Shanghai Men’s Health Study. Int. J. Epidemiol..

[20] Yuan S., Larsson S.C. (2020). An atlas on risk factors for type 2 diabetes: A wide-angled Mendelian randomisation study. Diabetologia.

[21] Stefanello N., Spanevello R.M., Passamonti S., Porciuncula L., Bonan C.D., Olabiyi A.A., Teixeira da Rocha J.B., Assmann C.E., Morsch V.M., Schetinger M.R.C. (2019). Coffee, caffeine, chlorogenic acid, and the purinergic system. Food Chem. Toxicol..

[22] Lane J.D., Feinglos M.N., Surwit R.S. (2008). Caffeine increases ambulatory glucose and postprandial responses in coffee drinkers with type 2 diabetes. Diabetes Care.

[23] Harpaz E., Tamir S., Weinstein A., Weinstein Y. (2017). The effect of caffeine on energy balance. J. Basic Clin. Physiol. Pharmacol..

[24] Keijzers G.B., De Galan B.E., Tack C.J., Smits P. (2002). Caffeine can decrease insulin sensitivity in humans. Diabetes Care.

[25] Dulloo A.G., Geissler C.A., Horton T., Collins A., Miller D.S. (1989). Normal caffeine consumption: Influence on thermogenesis and daily energy expenditure in lean and postobese human volunteers. Am. J. Clin. Nutr..

[26] Greer F., Hudson R., Ross R., Graham T. (2001). Caffeine ingestion decreases glucose disposal during a hyperinsulinemic-euglycemic clamp in sedentary humans. Diabetes.

[27] MacKenzie T., Comi R., Sluss P., Keisari R., Manwar S., Kim J., Larson R., Baron J.A. (2007). Metabolic and hormonal effects of caffeine: Randomized, double-blind, placebo-controlled crossover trial. Metabolism.

[28] Moisey L.L., Kacker S., Bickerton A.C., Robinson L.E., Graham T.E. (2008). Caffeinated coffee consumption impairs blood glucose homeostasis in response to high and low glycemic index meals in healthy men. Am. J. Clin. Nutr..

[29] Gavrieli A., Karfopoulou E., Kardatou E., Spyreli E., Fragopoulou E., Mantzoros C.S., Yannakoulia M. (2013). Effect of different amounts of coffee on dietary intake and appetite of normal-weight and overweight/obese individuals. Obesity.

[30] Rakvaag E., Dragsted L.O. (2016). Acute effects of light and dark roasted coffee on glucose tolerance: A randomized, controlled crossover trial in healthy volunteers. Eur. J. Nutr..

[31] Robertson T.M., Clifford M.N., Penson S., Chope G., Robertson M.D. (2015). A single serving of caffeinated coffee impairs postprandial glucose metabolism in overweight men. Br. J. Nutr..

[32] Shi X., Xue W., Liang S., Zhao J., Zhang X. (2016). Acute caffeine ingestion reduces insulin sensitivity in healthy subjects: A systematic review and meta-analysis. Nutr. J..

[33] Alperet D.J., Rebello S.A., Khoo E.Y., Tay Z., Seah S.S., Tai B.C., Tai E.S., Emady-Azar S., Chou C.J., Darimont C. (2020). The effect of coffee consumption on insulin sensitivity and other biological risk factors for type 2 diabetes: A randomized placebo-controlled trial. Am. J. Clin. Nutr..

[34] Ohnaka K., Ikeda M., Maki T., Okada T., Shimazoe T., Adachi M., Nomura M., Takayanagi R., Kono S. (2012). Effects of 16-week consumption of caffeinated and decaffeinated instant coffee on glucose metabolism in a randomized controlled trial. J. Nutr. Metab..

[35] Wedick N.M., Brennan A.M., Sun Q., Hu F.B., Mantzoros C.S., van Dam R.M. (2011). Effects of caffeinated and decaffeinated coffee on biological risk factors for type 2 diabetes: A randomized controlled trial. Nutr. J..

[36] Tuomilehto J. (2009). Nonpharmacologic therapy and exercise in the prevention of type 2 diabetes. Diabetes Care.

[37] Ding M., Bhupathiraju S.N., Chen M., van Dam R.M., Hu F.B. (2014). Caffeinated and decaffeinated coffee consumption and risk of type 2 diabetes: A systematic review and a dose-response meta-analysis. Diabetes Care.

[38] McCusker R.R., Fuehrlein B., Goldberger B.A., Gold M.S., Cone E.J. (2006). Caffeine content of decaffeinated coffee. J. Anal. Toxicol..

[39] Battram D.S., Arthur R., Weekes A., Graham T.E. (2006). The glucose intolerance induced by caffeinated coffee ingestion is less pronounced than that due to alkaloid caffeine in men. J. Nutr..

[40] Lecoultre V., Carrel G., Egli L., Binnert C., Boss A., MacMillan E.L., Kreis R., Boesch C., Darimont C., Tappy L. (2014). Coffee consumption attenuates short-term fructose-induced liver insulin resistance in healthy men. Am. J. Clin. Nutr..

[41] Johnston K.L., Clifford M.N., Morgan L.M. (2003). Coffee acutely modifies gastrointestinal hormone secretion and glucose tolerance in humans: Glycemic effects of chlorogenic acid and caffeine. Am. J. Clin. Nutr..

[42] Van Dijk A.E., Olthof M.R., Meeuse J.C., Seebus E., Heine R.J., van Dam R.M. (2009). Acute effects of decaffeinated coffee and the major coffee components chlorogenic acid and trigonelline on glucose tolerance. Diabetes Care.

[43] Reis C.E.G., Paiva C.L.R.D., Amato A.A., Lofrano-Porto A., Wassell S., Bluck L.J.C., Dorea J.G., da Costa T.H.M. (2018). Decaffeinated coffee improves insulin sensitivity in healthy men. Br. J. Nutr..

[44] Wong T.H.T., Wan J.M.F., Tse I.M.Y., Sit W.H., Louie J.C.Y. (2020). Consuming decaffeinated coffee with milk and sugar added before a high-glycaemic index meal improves postprandial glycaemic and insulinaemic responses in healthy adults. Br. J. Nutr..

[45] Olthof M.R., van Dijk A.E., Deacon C.F., Heine R.J., van Dam R.M. (2011). Acute effects of decaffeinated coffee and the major coffee components chlorogenic acid and trigonelline on incretin hormones. Nutr. Metab..

[46] Greenberg J.A., Owen D.R., Geliebter A. (2010). Decaffeinated coffee and glucose metabolism in young men. Diabetes Care.

[47] Hang D., Zeleznik O.A., He X., Guasch-Ferre M., Jiang X., Li J., Liang L., Eliassen A.H., Clish C.B., Chan A.T. (2020). Metabolomic Signatures of Long-term Coffee Consumption and Risk of Type 2 Diabetes in Women. Diabetes Care.

[48] Kuang A., Erlund I., Herder C., Westerhuis J.A., Tuomilehto J., Cornelis M.C. (2018). Lipidomic Response to Coffee Consumption. Nutrients.

[49] Favari C., Righetti L., Tassotti M., Gethings L.A., Martini D., Rosi A., Antonini M., Rubert J., Manach C., Dei C.A. (2021). Metabolomic Changes after Coffee Consumption: New Paths on the Block. Mol. Nutr. Food Res..

[50] Cornelis M.C., Erlund I., Michelotti G.A., Herder C., Westerhuis J.A., Tuomilehto J. (2018). Metabolomic response to coffee consumption: Application to a three-stage clinical trial. J. Intern. Med..

[51] Kuang A., Erlund I., Herder C., Westerhuis J.A., Tuomilehto J., Cornelis M.C. (2020). Targeted proteomic response to coffee consumption. Eur. J. Nutr..

[52] Seow W.J., Low D.Y., Pan W.C., Gunther S.H., Sim X., Torta F., Herr D.R., Kovalik J.P., Ching J., Khoo C.M. (2020). Coffee, Black Tea, and Green Tea Consumption in Relation to Plasma Metabolites in an Asian Population. Mol. Nutr. Food Res..

[53] Karadas O., Mese G., Ozcivici E. (2019). Cytotoxic Tolerance of Healthy and Cancerous Bone Cells to Anti-microbial Phenolic Compounds Depend on Culture Conditions. Appl. Biochem. Biotechnol..

[54] Costea T., Nagy P., Ganea C., Szollosi J., Mocanu M.M. (2019). Molecular Mechanisms and Bioavailability of Polyphenols in Prostate Cancer. Int. J. Mol. Sci..

[55] Kobayashi H., Murata M., Kawanishi S., Oikawa S. (2020). Polyphenols with Anti-Amyloid beta Aggregation Show Potential Risk of Toxicity Via Pro-Oxidant Properties. Int. J. Mol. Sci..

[56] Olson K.R., Briggs A., Devireddy M., Iovino N.A., Skora N.C., Whelan J., Villa B.P., Yuan X., Mannam V., Howard S. (2020). Green tea polyphenolic antioxidants oxidize hydrogen sulfide to thiosulfate and polysulfides: A possible new mechanism underpinning their biological action. Redox Biol..

[57] Murakami A. (2014). Dose-dependent functionality and toxicity of green tea polyphenols in experimental rodents. Arch. Biochem. Biophys..

[58] Oketch-Rabah H.A., Roe A.L., Rider C.V., Bonkovsky H.L., Giancaspro G.I., Navarro V., Paine M.F., Betz J.M., Marles R.J., Casper S. (2020). United States Pharmacopeia (USP) comprehensive review of the hepatotoxicity of green tea extracts. Toxicol. Rep..

[59] Leon-Gonzalez A.J., Auger C., Schini-Kerth V.B. (2015). Pro-oxidant activity of polyphenols and its implication on cancer chemoprevention and chemotherapy. Biochem. Pharmacol..

[60] Shaito A., Posadino A.M., Younes N., Hasan H., Halabi S., Alhababi D., Al-Mohannadi A., Abdel-Rahman W.M., Eid A.H., Nasrallah G.K. (2020). Potential Adverse Effects of Resveratrol: A Literature Review. Int. J. Mol. Sci..

[61] Tong R., Wu X., Liu Y., Liu Y., Zhou J., Jiang X., Zhang L., He X., Ma L. (2020). Curcumin-Induced DNA Demethylation in Human Gastric Cancer Cells Is Mediated by the DNA-Damage Response Pathway. Oxid. Med. Cell. Longev..

[62] Lotito S.B., Frei B. (2006). Consumption of flavonoid-rich foods and increased plasma antioxidant capacity in humans: Cause, consequence, or epiphenomenon?. Free Radic. Biol. Med..

[63] Lang R., Dieminger N., Beusch A., Lee Y.M., Dunkel A., Suess B., Skurk T., Wahl A., Hauner H., Hofmann T. (2013). Bioappearance and pharmacokinetics of bioactives upon coffee consumption. Anal. Bioanal. Chem..

[64] EFSA Panel on Food Additives and Nutrient Sources Added to Food (ANS) (2018). Scientific opinion on the safety of green tea catechins. EFSA J..

[65] EFSA Panel on Dietetic Products, Nitrition and Allergies NDA (2015). Scientific Opinion on the safety of caffeine. EFSA J..

[66] Kempf K., Kolb H., Gartner B., Bytof G., Stiebitz H., Lantz I., Lang R., Hofmann T., Martin S. (2015). Cardiometabolic effects of two coffee blends differing in content for major constituents in overweight adults: A randomized controlled trial. Eur. J. Nutr..

[67] Ludwig I.A., Clifford M.N., Lean M.E., Ashihara H., Crozier A. (2014). Coffee: Biochemistry and potential impact on health. Food Funct..

[68] Murakami A., Ohnishi K. (2012). Target molecules of food phytochemicals: Food science bound for the next dimension. Food Funct..

[69] Sirota R., Gibson D., Kohen R. (2015). The role of the catecholic and the electrophilic moieties of caffeic acid in Nrf2/Keap1 pathway activation in ovarian carcinoma cell lines. Redox Biol..

[70] Wei M., Zheng Z., Shi L., Jin Y., Ji L. (2018). Natural Polyphenol Chlorogenic Acid Protects Against Acetaminophen-Induced Hepatotoxicity by Activating ERK/Nrf2 Antioxidative Pathway. Toxicol. Sci..

[71] Liang N., Dupuis J.H., Yada R.Y., Kitts D.D. (2019). Chlorogenic acid isomers directly interact with Keap 1-Nrf2 signaling in Caco-2 cells. Mol. Cell. Biochem..

[72] Ciolino H.P., Daschner P.J., Yeh G.C. (1999). Dietary flavonols quercetin and kaempferol are ligands of the aryl hydrocarbon receptor that affect CYP1A1 transcription differentially. Biochem. J..

[73] Kalthoff S., Ehmer U., Freiberg N., Manns M.P., Strassburg C.P. (2010). Coffee induces expression of glucuronosyltransferases by the aryl hydrocarbon receptor and Nrf2 in liver and stomach. Gastroenterology.

[74] Ishikawa T., Takahashi S., Morita K., Okinaga H., Teramoto T. (2014). Induction of AhR-mediated gene transcription by coffee. PLoS ONE.

[75] Safe S., Jin U.H., Park H., Chapkin R.S., Jayaraman A. (2020). Aryl Hydrocarbon Receptor (AHR) Ligands as Selective AHR Modulators (SAhRMs). Int. J. Mol. Sci..

[76] Altieri F., Cairone F., Giamogante F., Carradori S., Locatelli M., Chichiarelli S., Cesa S. (2019). Influence of Ellagitannins Extracted by Pomegranate Fruit on Disulfide Isomerase PDIA3 Activity. Nutrients.

[77] Islam B., Sharma C., Adem A., Aburawi E., Ojha S. (2015). Insight into the mechanism of polyphenols on the activity of HMGR by molecular docking. Drug Des. Devel. Ther..

[78] Liu G., Shi A., Wang N., Li M., He X., Yin C., Tu Q., Shen X., Tao Y., Wang Q. (2020). Polyphenolic Proanthocyanidin-B2 suppresses proliferation of liver cancer cells and hepatocellular carcinogenesis through directly binding and inhibiting AKT activity. Redox Biol..

[79] Vachali P.P., Li B., Besch B.M., Bernstein P.S. (2016). Protein-Flavonoid Interaction Studies by a Taylor Dispersion Surface Plasmon Resonance (SPR) Technique: A Novel Method to Assess Biomolecular Interactions. Biosensors.

[80] Hu W.H., Dai D.K., Zheng B.Z., Duan R., Dong T.T., Qin Q.W., Tsim K.W. (2020). Piceatannol, a Natural Analog of Resveratrol, Exerts Anti-angiogenic Efficiencies by Blockage of Vascular Endothelial Growth Factor Binding to Its Receptor. Molecules.

[81] Aranaz P., Navarro-Herrera D., Zabala M., Migueliz I., Romo-Hualde A., Lopez-Yoldi M., Martinez J.A., Vizmanos J.L., Milagro F.I., Gonzalez-Navarro C.J. (2019). Phenolic Compounds Inhibit 3T3-L1 Adipogenesis Depending on the Stage of Differentiation and Their Binding Affinity to PPARgamma. Molecules.

[82] Wang D., Ho L., Faith J., Ono K., Janle E.M., Lachcik P.J., Cooper B.R., Jannasch A.H., D’Arcy B.R., Williams B.A. (2015). Role of intestinal microbiota in the generation of polyphenol-derived phenolic acid mediated attenuation of Alzheimer’s disease beta-amyloid oligomerization. Mol. Nutr. Food Res..

[83] Dubey R., Patil K., Dantu S.C., Sardesai D.M., Bhatia P., Malik N., Acharya J.D., Sarkar S., Ghosh S., Chakrabarti R. (2019). Azadirachtin inhibits amyloid formation, disaggregates pre-formed fibrils and protects pancreatic beta-cells from human islet amyloid polypeptide/amylin-induced cytotoxicity. Biochem. J..

[84] Jahic A., Tusek Z.M., Pintar S., Berbic S., Zerovnik E. (2020). The effect of three polyphenols and some other antioxidant substances on amyloid fibril formation by Human cystatin C. Neurochem. Int..

[85] Araujo A.R., Reis R.L., Pires R.A. (2020). Natural Polyphenols as Modulators of the Fibrillization of Islet Amyloid Polypeptide. Adv. Exp. Med. Biol..

[86] Chaari A. (2020). Inhibition of human islet amyloid polypeptide aggregation and cellular toxicity by oleuropein and derivatives from olive oil. Int. J. Biol. Macromol..

[87] Chaari A., Abdellatif B., Nabi F., Khan R.H. (2020). Date palm (Phoenix dactylifera L.) fruit’s polyphenols as potential inhibitors for human amylin fibril formation and toxicity in type 2 diabetes. Int. J. Biol. Macromol..

[88] Charlton A.J., Baxter N.J., Khan M.L., Moir A.J., Haslam E., Davies A.P., Williamson M.P. (2002). Polyphenol/peptide binding and precipitation. J. Agric. Food Chem..

[89] Sinisi V., Forzato C., Cefarin N., Navarini L., Berti F. (2015). Interaction of chlorogenic acids and quinides from coffee with human serum albumin. Food Chem..

[90] Berti F., Navarini L., Guercia E., Oreski A., Gasparini A., Scoltock J., Forzato C. (2020). Interaction of the Coffee Diterpenes Cafestol and 16-O-Methyl-Cafestol Palmitates with Serum Albumins. Int. J. Mol. Sci..

[91] Tung W.C., Rizzo B., Dabbagh Y., Saraswat S., Romanczyk M., Codorniu-Hernandez E., Rebollido-Rios R., Needs P.W., Kroon P.A., Rakotomanomana N. (2020). Polyphenols bind to low density lipoprotein at biologically relevant concentrations that are protective for heart disease. Arch. Biochem. Biophys..

[92] Kolb H., Kempf K., Martin S. (2020). Health Effects of Coffee: Mechanism Unraveled?. Nutrients.

[93] Shao B., Mao L., Shao J., Huang C.H., Qin L., Huang R., Sheng Z.G., Cao D., Zhang Z.Q., Lin L. (2020). Mechanism of synergistic DNA damage induced by caffeic acid phenethyl ester (CAPE) and Cu(II): Competitive binding between CAPE and DNA with Cu(II)/Cu(I). Free Radic. Biol. Med..

[94] Wang Z., Zhai X., Sun Y., Yin C., Yang E., Wang W., Sun D. (2020). Antibacterial activity of chlorogenic acid-loaded SiO2 nanoparticles caused by accumulation of reactive oxygen species. Nanotechnology.

[95] Kanner J. (2020). Polyphenols by Generating H2O2, Affect Cell Redox Signaling, Inhibit PTPs and Activate Nrf2 Axis for Adaptation and Cell Surviving: In Vitro, In Vivo and Human Health. Antioxidants.

[96] Labbadia J., Brielmann R.M., Neto M.F., Lin Y.F., Haynes C.M., Morimoto R.I. (2017). Mitochondrial Stress Restores the Heat Shock Response and Prevents Proteostasis Collapse during Aging. Cell Rep..

[97] Boncler M., Golanski J., Lukasiak M., Redzynia M., Dastych J., Watala C. (2017). A new approach for the assessment of the toxicity of polyphenol-rich compounds with the use of high content screening analysis. PLoS ONE.

[98] Franco R., Navarro G., Martinez-Pinilla E. (2019). Hormetic and Mitochondria-Related Mechanisms of Antioxidant Action of Phytochemicals. Antioxidants.

[99] Calabrese V., Cornelius C., Dinkova-Kostova A.T., Iavicoli I., Di P.R., Koverech A., Cuzzocrea S., Rizzarelli E., Calabrese E.J. (2012). Cellular stress responses, hormetic phytochemicals and vitagenes in aging and longevity. Biochim. Biophys. Acta.

[100] Naoi M., Wu Y., Shamoto-Nagai M., Maruyama W. (2019). Mitochondria in Neuroprotection by Phytochemicals: Bioactive Polyphenols Modulate Mitochondrial Apoptosis System, Function and Structure. Int. J. Mol. Sci..

[101] Rothhammer V., Quintana F.J. (2019). The aryl hydrocarbon receptor: An environmental sensor integrating immune responses in health and disease. Nat. Rev. Immunol..

[102] Kalthoff S., Ehmer U., Freiberg N., Manns M.P., Strassburg C.P. (2010). Interaction between oxidative stress sensor Nrf2 and xenobiotic-activated aryl hydrocarbon receptor in the regulation of the human phase II detoxifying UDP-glucuronosyltransferase 1A10. J. Biol. Chem..

[103] Kohle C., Bock K.W. (2007). Coordinate regulation of Phase I and II xenobiotic metabolisms by the Ah receptor and Nrf2. Biochem. Pharmacol..

[104] Grahame H.D. (2014). AMP-activated protein kinase: A key regulator of energy balance with many roles in human disease. J. Intern. Med..

[105] Salminen A., Kauppinen A., Kaarniranta K. (2016). AMPK/Snf1 signaling regulates histone acetylation: Impact on gene expression and epigenetic functions. Cell Signal..

[106] Iside C., Scafuro M., Nebbioso A., Altucci L. (2020). SIRT1 Activation by Natural Phytochemicals: An Overview. Front. Pharmacol..

[107] Lei L., Chai Y., Lin H., Chen C., Zhao M., Xiong W., Zhuang J., Fan X. (2020). Dihydroquercetin Activates AMPK/Nrf2/HO-1 Signaling in Macrophages and Attenuates Inflammation in LPS-Induced Endotoxemic Mice. Front. Pharmacol..

[108] Boettler U., Sommerfeld K., Volz N., Pahlke G., Teller N., Somoza V., Lang R., Hofmann T., Marko D. (2011). Coffee constituents as modulators of Nrf2 nuclear translocation and ARE (EpRE)-dependent gene expression. J. Nutr. Biochem..

[109] Shi A., Shi H., Wang Y., Liu X., Cheng Y., Li H., Zhao H., Wang S., Dong L. (2018). Activation of Nrf2 pathway and inhibition of NLRP3 inflammasome activation contribute to the protective effect of chlorogenic acid on acute liver injury. Int. Immunopharmacol..

[110] Fratantonio D., Speciale A., Canali R., Natarelli L., Ferrari D., Saija A., Virgili F., Cimino F. (2017). Low nanomolar caffeic acid attenuates high glucose-induced endothelial dysfunction in primary human umbilical-vein endothelial cells by affecting NF-kappaB and Nrf2 pathways. Biofactors.

[111] Sauer T., Raithel M., Kressel J., Munch G., Pischetsrieder M. (2013). Activation of the transcription factor Nrf2 in macrophages, Caco-2 cells and intact human gut tissue by Maillard reaction products and coffee. Amino Acids.

[112] Balstad T.R., Carlsen H., Myhrstad M.C., Kolberg M., Reiersen H., Gilen L., Ebihara K., Paur I., Blomhoff R. (2011). Coffee, broccoli and spices are strong inducers of electrophile response element-dependent transcription in vitro and in vivo—Studies in electrophile response element transgenic mice. Mol. Nutr. Food Res..

[113] Higgins L.G., Cavin C., Itoh K., Yamamoto M., Hayes J.D. (2008). Induction of cancer chemopreventive enzymes by coffee is mediated by transcription factor Nrf2. Evidence that the coffee-specific diterpenes cafestol and kahweol confer protection against acrolein. Toxicol. Appl. Pharmacol..

[114] Ren Y., Wang C., Xu J., Wang S. (2019). Cafestol and Kahweol: A Review on Their Bioactivities and Pharmacological Properties. Int. J. Mol. Sci..

[115] Fouzder C., Mukhuty A., Mukherjee S., Malick C., Kundu R. (2021). Trigonelline inhibits Nrf2 via EGFR signalling pathway and augments efficacy of Cisplatin and Etoposide in NSCLC cells. Toxicol. Vitro.

[116] Boettler U., Volz N., Pahlke G., Teller N., Kotyczka C., Somoza V., Stiebitz H., Bytof G., Lantz I., Lang R. (2011). Coffees rich in chlorogenic acid or N-methylpyridinium induce chemopreventive phase II-enzymes via the Nrf2/ARE pathway in vitro and in vivo. Mol. Nutr. Food Res..

[117] Zamora-Ros R., Rothwell J.A., Scalbert A., Knaze V., Romieu I., Slimani N., Fagherazzi G., Perquier F., Touillaud M., Molina-Montes E. (2013). Dietary intakes and food sources of phenolic acids in the European Prospective Investigation into Cancer and Nutrition (EPIC) study. Br. J. Nutr..

[118] Taguchi C., Fukushima Y., Kishimoto Y., Suzuki-Sugihara N., Saita E., Takahashi Y., Kondo K. (2015). Estimated Dietary Polyphenol Intake and Major Food and Beverage Sources among Elderly Japanese. Nutrients.

[119] Zamora-Ros R., Knaze V., Rothwell J.A., Hemon B., Moskal A., Overvad K., Tjonneland A., Kyro C., Fagherazzi G., Boutron-Ruault M.C. (2016). Dietary polyphenol intake in Europe: The European Prospective Investigation into Cancer and Nutrition (EPIC) study. Eur. J. Nutr..

[120] Cavin C., Marin-Kuan M., Langouet S., Bezencon C., Guignard G., Verguet C., Piguet D., Holzhauser D., Cornaz R., Schilter B. (2008). Induction of Nrf2-mediated cellular defenses and alteration of phase I activities as mechanisms of chemoprotective effects of coffee in the liver. Food Chem. Toxicol..

[121] Paur I., Balstad T.R., Blomhoff R. (2010). Degree of roasting is the main determinant of the effects of coffee on NF-kappaB and EpRE. Free Radic. Biol. Med..

[122] Salomone F., Li V.G., Vitaglione P., Morisco F., Fogliano V., Zappala A., Palmigiano A., Garozzo D., Caporaso N., D’Argenio G. (2014). Coffee enhances the expression of chaperones and antioxidant proteins in rats with nonalcoholic fatty liver disease. Transl. Res..

[123] Vicente S.J., Ishimoto E.Y., Torres E.A. (2014). Coffee modulates transcription factor Nrf2 and highly increases the activity of antioxidant enzymes in rats. J. Agric. Food Chem..

[124] Volz N., Boettler U., Winkler S., Teller N., Schwarz C., Bakuradze T., Eisenbrand G., Haupt L., Griffiths L.R., Stiebitz H. (2012). Effect of coffee combining green coffee bean constituents with typical roasting products on the Nrf2/ARE pathway in vitro and in vivo. J. Agric. Food Chem..

[125] Priftis A., Angeli-Terzidou A.E., Veskoukis A.S., Spandidos D.A., Kouretas D. (2018). Cellspecific and roastingdependent regulation of the Keap1/Nrf2 pathway by coffee extracts. Mol. Med. Rep..

[126] Salomone F., Galvano F., Li V.G. (2017). Molecular Bases Underlying the Hepatoprotective Effects of Coffee. Nutrients.

[127] Alferink L.J.M., Kiefte-de Jong J.C., Darwish M.S. (2018). Potential Mechanisms Underlying the Role of Coffee in Liver Health. Semin. Liver Dis..

[128] Farias-Pereira R., Park C.S., Park Y. (2019). Mechanisms of action of coffee bioactive components on lipid metabolism. Food Sci. Biotechnol..

[129] Meex R.C.R., Blaak E.E. (2021). Mitochondrial Dysfunction is a Key Pathway that Links Saturated Fat Intake to the Development and Progression of NAFLD. Mol. Nutr. Food Res..

[130] Brandt A., Nier A., Jin C.J., Baumann A., Jung F., Ribas V., Garcia-Ruiz C., Fernandez-Checa J.C., Bergheim I. (2019). Consumption of decaffeinated coffee protects against the development of early non-alcoholic steatohepatitis: Role of intestinal barrier function. Redox Biol..

[131] Vitaglione P., Mazzone G., Lembo V., D’Argenio G., Rossi A., Guido M., Savoia M., Salomone F., Mennella I., De F.F. (2019). Coffee prevents fatty liver disease induced by a high-fat diet by modulating pathways of the gut-liver axis. J. Nutr. Sci..

[132] Chen Y.P., Lu F.B., Hu Y.B., Xu L.M., Zheng M.H., Hu E.D. (2019). A systematic review and a dose-response meta-analysis of coffee dose and nonalcoholic fatty liver disease. Clin. Nutr..

[133] Hayat U., Siddiqui A.A., Okut H., Afroz S., Tasleem S., Haris A. (2021). The effect of coffee consumption on the non-alcoholic fatty liver disease and liver fibrosis: A meta-analysis of 11 epidemiological studies. Ann. Hepatol..

[134] Weir G.C., Gaglia J., Bonner-Weir S. (2020). Inadequate beta-cell mass is essential for the pathogenesis of type 2 diabetes. Lancet Diabetes Endocrinol..

[135] Esser N., Utzschneider K.M., Kahn S.E. (2020). Early beta cell dysfunction vs insulin hypersecretion as the primary event in the pathogenesis of dysglycaemia. Diabetologia.

[136] Kolb H., Stumvoll M., Kramer W., Kempf K., Martin S. (2018). Insulin translates unfavourable lifestyle into obesity. BMC Med..

[137] Marselli L., Piron A., Suleiman M., Colli M.L., Yi X., Khamis A., Carrat G.R., Rutter G.A., Bugliani M., Giusti L. (2020). Persistent or Transient Human beta Cell Dysfunction Induced by Metabolic Stress: Specific Signatures and Shared Gene Expression with Type 2 Diabetes. Cell Rep..

[138] Taylor R., Al-Mrabeh A., Sattar N. (2019). Understanding the mechanisms of reversal of type 2 diabetes. Lancet Diabetes Endocrinol..

[139] Accili D., Talchai S.C., Kim-Muller J.Y., Cinti F., Ishida E., Ordelheide A.M., Kuo T., Fan J., Son J. (2016). When beta-cells fail: Lessons from dedifferentiation. Diabetes Obes. Metab..

[140] Raleigh D., Zhang X., Hastoy B., Clark A. (2017). The beta-cell assassin: IAPP cytotoxicity. J Mol Endocrinol.

[141] Arunagiri A., Haataja L., Pottekat A., Pamenan F., Kim S., Zeltser L.M., Paton A.W., Paton J.C., Tsai B., Itkin-Ansari P. (2019). Proinsulin misfolding is an early event in the progression to type 2 diabetes. Elife.

[142] Carrasco-Pozo C., Tan K.N., Gotteland M., Borges K. (2017). Sulforaphane Protects against High Cholesterol-Induced Mitochondrial Bioenergetics Impairments, Inflammation, and Oxidative Stress and Preserves Pancreatic beta-Cells Function. Oxid. Med. Cell. Longev..

[143] Li C.G., Ni C.L., Yang M., Tang Y.Z., Li Z., Zhu Y.J., Jiang Z.H., Sun B., Li C.J. (2018). Honokiol protects pancreatic beta cell against high glucose and intermittent hypoxia-induced injury by activating Nrf2/ARE pathway in vitro and in vivo. Biomed. Pharmacother..

[144] Schultheis J., Beckmann D., Mulac D., Muller L., Esselen M., Dufer M. (2019). Nrf2 Activation Protects Mouse Beta Cells from Glucolipotoxicity by Restoring Mitochondrial Function and Physiological Redox Balance. Oxid. Med. Cell. Longev..

[145] Moens C., Bensellam M., Himpe E., Muller C.J.F., Jonas J.C., Bouwens L. (2020). Aspalathin Protects Insulin-Producing beta Cells against Glucotoxicity and Oxidative Stress-Induced Cell Death. Mol. Nutr. Food Res..

[146] Ganesan K., Ramkumar K.M., Xu B. (2020). Vitexin restores pancreatic beta-cell function and insulin signaling through Nrf2 and NF-kappaB signaling pathways. Eur. J. Pharmacol..

[147] Baumel-Alterzon S., Katz L.S., Brill G., Garcia-Ocana A., Scott D.K. (2021). Nrf2: The Master and Captain of Beta Cell Fate. Trends Endocrinol. Metab..

[148] Fu J., Zheng H., Wang H., Yang B., Zhao R., Lu C., Liu Z., Hou Y., Xu Y., Zhang Q. (2015). Protective Role of Nuclear Factor E2-Related Factor 2 against Acute Oxidative Stress-Induced Pancreatic beta -Cell Damage. Oxid. Med. Cell. Longev..

[149] Kumar A., Katz L.S., Schulz A.M., Kim M., Honig L.B., Li L., Davenport B., Homann D., Garcia-Ocana A., Herman M.A. (2018). Activation of Nrf2 Is Required for Normal and ChREBPalpha-Augmented Glucose-Stimulated beta-Cell Proliferation. Diabetes.

[150] Uruno A., Furusawa Y., Yagishita Y., Fukutomi T., Muramatsu H., Negishi T., Sugawara A., Kensler T.W., Yamamoto M. (2013). The Keap1-Nrf2 system prevents onset of diabetes mellitus. Mol. Cell. Biol..

[151] Watanabe S., Takahashi T., Ogawa H., Uehara H., Tsunematsu T., Baba H., Morimoto Y., Tsuneyama K. (2017). Daily Coffee Intake Inhibits Pancreatic Beta Cell Damage and Nonalcoholic Steatohepatitis in a Mouse Model of Spontaneous Metabolic Syndrome, Tsumura-Suzuki Obese Diabetic Mice. Metab. Syndr. Relat. Disord..

[152] Ciaramelli C., Palmioli A., De L.A., Colombo L., Sala G., Riva C., Zoia C.P., Salmona M., Airoldi C. (2018). NMR-driven identification of anti-amyloidogenic compounds in green and roasted coffee extracts. Food Chem..

[153] Fukuyama K., Kakio S., Nakazawa Y., Kobata K., Funakoshi-Tago M., Suzuki T., Tamura H. (2018). Roasted Coffee Reduces beta-Amyloid Production by Increasing Proteasomal beta-Secretase Degradation in Human Neuroblastoma SH-SY5Y Cells. Mol. Nutr. Food Res..

[154] Ishida K., Misawa K., Nishimura H., Hirata T., Yamamoto M., Ota N. (2020). 5-Caffeoylquinic Acid Ameliorates Cognitive Decline and Reduces Abeta Deposition by Modulating Abeta Clearance Pathways in APP/PS2 Transgenic Mice. Nutrients.

[155] Costes S., Langen R., Gurlo T., Matveyenko A.V., Butler P.C. (2013). beta-Cell failure in type 2 diabetes: A case of asking too much of too few?. Diabetes.

[156] Bakuradze T., Lang R., Hofmann T., Schipp D., Galan J., Eisenbrand G., Richling E. (2016). Coffee consumption rapidly reduces background DNA strand breaks in healthy humans: Results of a short-term repeated uptake intervention study. Mol. Nutr. Food Res..

[157] Kotyczka C., Boettler U., Lang R., Stiebitz H., Bytof G., Lantz I., Hofmann T., Marko D., Somoza V. (2011). Dark roast coffee is more effective than light roast coffee in reducing body weight, and in restoring red blood cell vitamin E and glutathione concentrations in healthy volunteers. Mol. Nutr. Food Res..

